# Exploring the role of medicinal plants and nanotechnology in the management of polycystic ovarian syndrome (PCOS): a scoping review

**DOI:** 10.1007/s00210-026-05425-3

**Published:** 2026-05-14

**Authors:** Obukowho Benson Ichipi, Samuel Oluwaseun Olojede, Sodiq Kolawole Lawal, Onyemaechi Okpara Azu, Edwin Coleridge Naidu

**Affiliations:** 1 Discipline of Clinical Anatomy, School of Laboratory Medicine and Medical Sciences, Nelson R Mandela School of Medicine, University of KwaZulu-Natal, Durban, South Africa; 2 Division of Human Anatomy, School of Biomedical Sciences, Faculty of Medicine and Health Sciences, Walter Sisulu University, Nelson Mandela Drive, 5099 Mthatha South Africa; 3 School of Nursing, Faculty of Health Sciences, University of Botswana, Private Bag UB 0022, Plot 4775, Notwane Road, Gaborone, Botswana; 4 Department of Medical Biosciences, University of the Western Cape, Bellville, South Africa

**Keywords:** Polycystic ovarian syndrome (PCOS), Plant-derived metallic nanoparticles, Green synthesis, Rodent PCOS model, Preclinical evidence

## Abstract

Polycystic ovary syndrome (PCOS) is a complex endocrine disorder characterized by heterogeneous symptoms and is commonly managed with conventional medications that often exhibit variable efficacy and undesirable side effects. Given these limitations, nanotechnology, particularly plant-derived metallic nanoparticles, offers novel strategies to enhance phytochemical delivery, stability, and bioavailability. Accordingly, this scoping review synthesizes preclinical evidence (2015–2025) on plant-derived metallic nanoparticles for PCOS, evaluating therapeutic outcomes, safety, and mechanisms. In line with PRISMA-ScR guidance, records were identified from PubMed, Scopus, and ScienceDirect (*n* = 375), duplicates were removed (*n* = 51), and 324 records underwent screening. Three hundred twenty records were excluded based on title/abstract screening, and 4 reports were retrieved from databases for eligibility assessment. Together with 1 additional study from Google Scholar, 5 rodent studies were included. Reporting quality and risk of bias were assessed descriptively using selected ARRIVE- and SYRCLE-based domains. Overall, the included studies evaluated *cinnamon*-derived silver nanoparticles, selenium nanoparticles synthesized using *Corchorus olitorius*, silver nanoparticles synthesized from aqueous extract of fresh *asparagus* stems, and silver nanoparticles prepared using *fenugreek* aqueous extract. Across the included studies, these interventions normalized reproductive hormones, restored estrous cyclicity and fertility, reduced ovarian cystic changes, and lowered inflammatory markers (TNF-α, IL-6, IL-10, IL-17, IL-18) without observable toxicity. Nevertheless, clinical translation remains pending due to the absence of long-term safety data and well-structured human clinical trials. Future research should prioritize mechanistic validation, long-term toxicological assessment, formulation standardization, and rigorously designed clinical trials.

**Supplementary Information:**

The online version contains supplementary material available at 10.1007/s00210-026-05425-3.

## Introduction

Polycystic ovarian syndrome (PCOS) is one of the most prevalent neuro-endocrine disorders, impacting approximately 6–20% of women of reproductive age globally (Azziz et al. 2016; Escobar-Morreale 2018; Teede et al. 2023; Akintola et al. 2024; Shi et al. 2024). Depending on the diagnostic framework, global PCOS prevalence ranges from approximately 6.6% by NIH criteria to 10.9% by Rotterdam criteria, with the AE-PCOS algorithm yielding about 10.6% (ESHRE/ASRM 2004; Bozdag et al. 2016; Amiri et al. 2025). It is characterized by hyperandrogenism, chronic anovulation, polycystic ovaries, and metabolic disturbances such as insulin resistance (Azziz et al. 2016; Gibson-Helm et al. 2017; Paixão et al. 2017; Akintola et al. 2024). Consequently, PCOS manifests with diverse symptoms including menstrual irregularities, infertility, hirsutism, acne, obesity, and an increased risk of type 2 diabetes and cardiovascular disease (Toulis et al. 2011; Chandrasekaran and Sagili 2018; Wekker et al. 2020; Barber and Franks 2021; Melin et al. 2023; Shi et al. 2024).

**Fig. 1:**
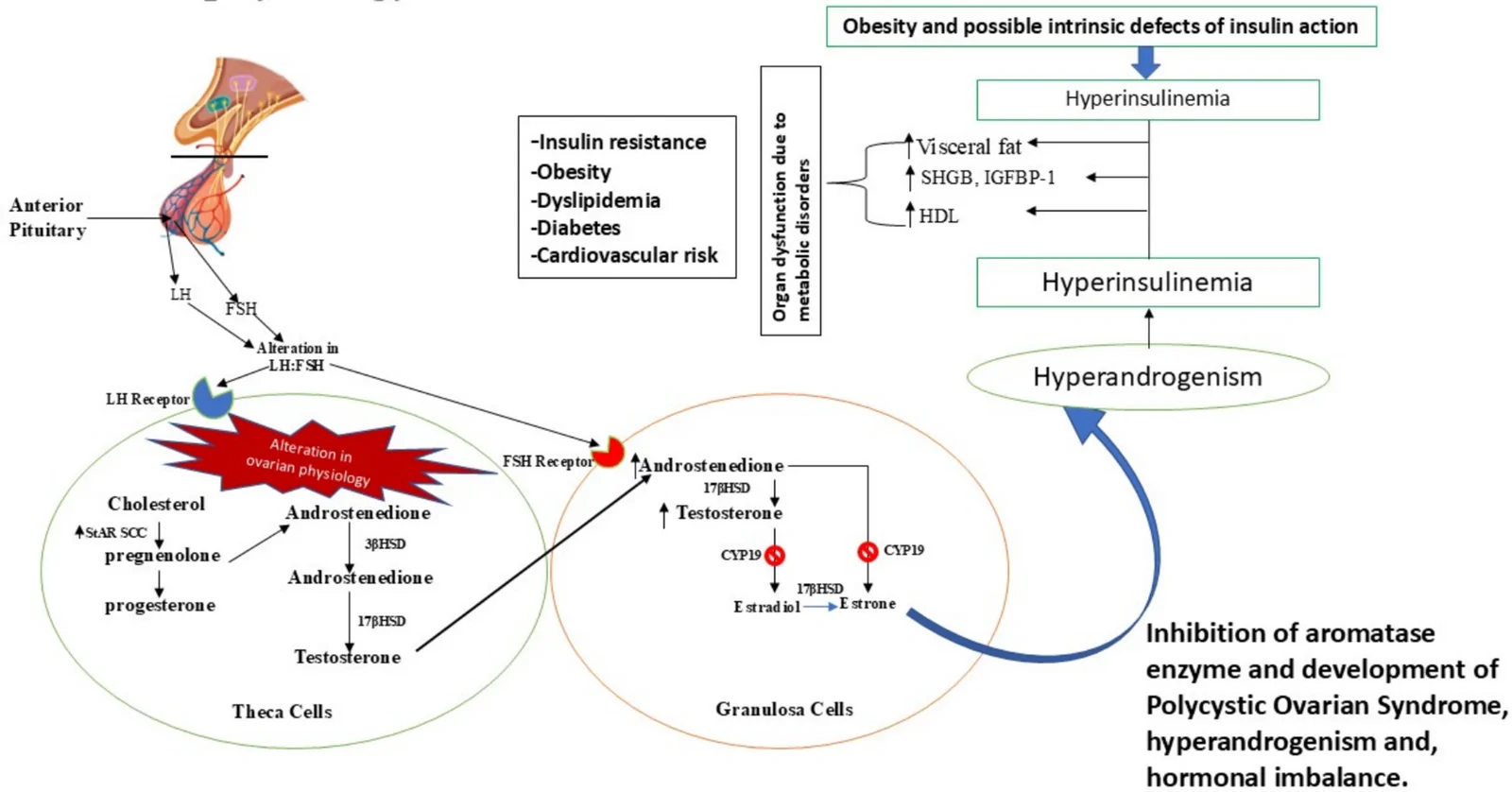
Schematic overview of key pathophysiological pathways implicated in PCOS. LH, luteinizing hormone; FSH, follicle-stimulating hormone; LHR (LH receptor), luteinizing hormone receptor; FSH receptor, follicle-stimulating hormone receptor; StAR, steroidogenic acute regulatory protein; SCC (P450scc/CYP11A1), cholesterol side-chain cleavage enzyme; 3β-HSD, 3-beta-hydroxysteroid dehydrogenase; 17β-HSD, 17-beta-hydroxysteroid dehydrogenase; CYP19, aromatase (cytochrome P450 family 19; CYP19A1); SHBG, sex hormone-binding globulin; IGFBP-1, insulin-like growth factor binding protein-1; HDL, high-density lipoprotein

Mechanistically, the pathophysiology of PCOS involves dysregulation of the hypothalamic–pituitary–ovarian (HPO) axis, characterized by heightened gonadotropin-releasing hormone (GnRH) activity and luteinizing hormone (LH) secretion, which drives excessive ovarian androgen synthesis (Rosenfield and Ehrmann 2016; Barber and Franks 2021; Akintola et al. 2024; Bahreiny et al. 2024; Shi et al. 2024). Furthermore, concurrent insulin resistance and compensatory hyperinsulinemia further amplify androgen production, while chronic low-grade inflammation and oxidative stress disrupt normal ovarian function (Gonzalez 2012; Murri et al. 2013; Abdallah et al. 2023). These interacting endocrine–metabolic pathways are summarized in Fig. 1.

Conventional therapies for PCOS include lifestyle modifications and pharmacological interventions such as oral contraceptives, insulin-sensitizers (metformin), and anti-androgens (Legro et al. 2013; Akre et al. 2022; Rashid et al. 2022; Teede et al. 2023). However, these treatments can be suboptimal for many patients and are associated with side effects, such as gastrointestinal discomfort with metformin and thrombotic risk with oral contraceptives (Fonseka et al. 2020; Melin et al. 2023; Shi et al. 2024). Moreover, the heterogeneity of PCOS symptoms often necessitates combination therapy, yet a single effective cure is lacking (Alwan and Al-Saeed 2023; Kelly et al. 2025).

In addition to conventional treatments, herbal medicine has a long history in managing reproductive and metabolic disorders (Kwon et al. 2020; Manouchehri et al. 2023; Sharma et al. 2025). Nevertheless, many phytochemicals exhibit poor water solubility, instability, or low bioavailability when administered orally (Lopresti 2018; Shi et al. 2024; Zhao et al. 2025). In this context, nanotechnology may provide a complementary approach by loading or synthesizing plant compounds into nanoparticle formulations to protect bioactive compounds from degradation, enable controlled release, enhance transport across biological barriers and tissue uptake, and improve bioavailability, stability, targeted delivery, and therapeutic specificity (Blanco et al. 2015; Alwan and Al-Saeed 2021, 2023; Mitchell et al. 2021; Akintola et al. 2024; Naidu et al. 2024; Shi et al. 2024; Maddirevula et al. 2025).

**Fig. 2:**
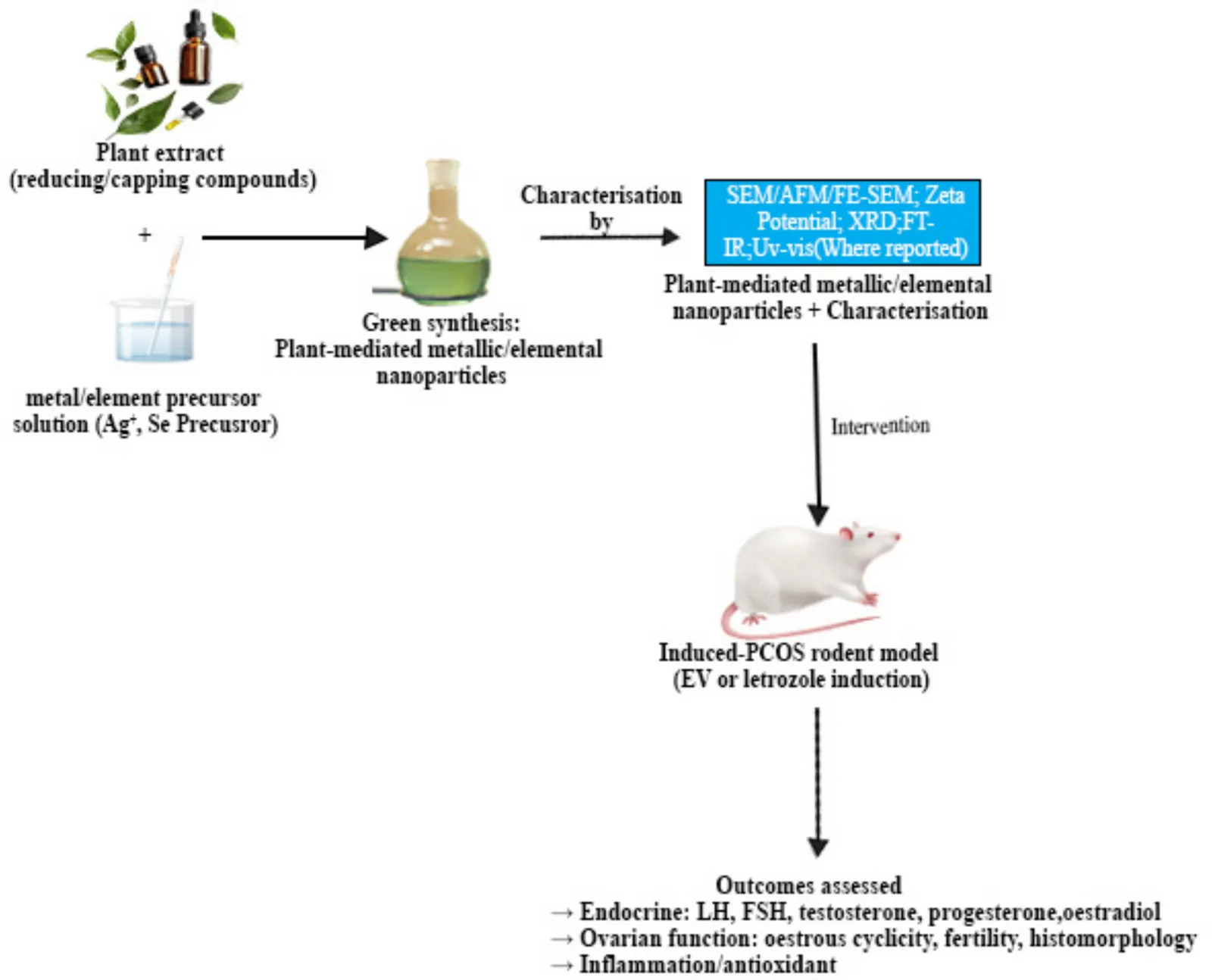
Overview of the green-synthesis workflow for plant-mediated nanoparticles and the key PCOS outcomes assessed in included animal studies. Created by the authors using and adapting Freepik elements (brgfx 2023; Freepik 2026; Macrovector 2020; Tohamina 2025)

Specifically, in the context of PCOS, nanocarriers may be used to deliver herbal extracts or natural compounds to ovarian tissue and/or modulate systemic metabolic pathways with fewer side effects (Shi et al. 2024). Accordingly, the application of diverse nanomaterials in PCOS management has gained considerable attention and this includes metallic nanoparticles (silver, selenium) produced via green synthesis using plant extracts (Ashkar et al. 2020; Miere et al. 2022; Alwan and Al-Saeed 2023; Akintola et al. 2024; Al-Bayati et al. 2024; Shi et al. 2024). An overview of the green-synthesis workflow and outcomes evaluated in this review is presented in Fig. 2.

Accordingly, this scoping review focuses on plant-mediated metallic/elemental nanoparticles produced via green synthesis using plant extracts, evaluated in induced-PCOS rodent models. In contrast to broader PCOS nanomedicine reviews spanning multiple nanocarrier classes and non-plant formulations, this delimitation allows targeted comparison of endocrine and inflammatory endpoints, together with reported safety observations (Alwan and Al-Saeed 2021; Akintola et al. 2024; Shi et al. 2024). Relative to liposomes and synthetic polymer platforms, plant-mediated metallic/elemental nanoparticles may offer bio-functional phytochemical-derived surface capping and streamlined synthesis routes; however, reproducibility, batch-to-batch variability, and standardization remain key limitations (Al-Bayati et al. 2024; Shi et al. 2024). Overall, the current evidence base is limited and preclinical, and conclusions are framed conservatively.

Accordingly, this scoping review addressed the following question: What preclinical evidence exists regarding the therapeutic effects of plant-mediated metallic/elemental nanoparticles in induced-PCOS rodent models?

## Material and methods

This scoping review was conducted following Arksey and O’Malley’s framework and reported in accordance with the PRISMA-ScR (Preferred Reporting Items for Systematic Reviews and Meta-Analyses extension for Scoping Reviews) guidelines (Arksey and O'Malley 2005; Levac et al. 2010; Tricco et al. 2018).

### Literature search strategy

A literature search was performed to identify relevant studies published between 2015 and 2025. The search was conducted in three electronic databases (PubMed, Scopus, and ScienceDirect), with Google Scholar searched via Publish or Perish as an additional source. Search terms were entered manually in the advanced search interfaces using Boolean operators (AND, OR), parentheses for term grouping, quotation marks for phrase searching, and the wildcard symbol (*) where applicable. The searches were restricted to English-language publications. The full database-specific search strings and applied limits are provided in Supplementary Table S1. Because the database strategy was designed to be sensitive, the final restriction to plant-extract-mediated metallic/elemental nanoparticles and induced-PCOS rodent models was applied during screening and full-text eligibility assessment.

### Study selection criteria

Following duplicate removal, titles/abstracts and full texts were screened independently by two reviewers against predefined eligibility criteria. Discrepancies were resolved by discussion to achieve consensus; where consensus was not reached, a third reviewer adjudicated, in line with scoping-review screening guidance. Studies were eligible if they met all of the following:

Inclusion criteria

- Population: used an induced-PCOS rodent model (rat or mouse).
- Intervention: evaluated plant-extract–mediated metallic/elemental nanoparticles (i.e., nanoparticles synthesized via plant extracts/green method) as the therapeutic exposure.
- Outcomes: reported PCOS-relevant endpoints (reproductive hormones, ovarian morphology/histology, metabolic indices, inflammatory/oxidative markers).
- Study type: original preclinical therapeutic studies.
- Nanoparticle characterization: reported core physicochemical features (size and morphology, Ultraviolet–visible spectroscopy (UV–Vis) and/or other characterization where available).

Exclusion criteria

- Review articles, scoping reviews, systematic reviews, meta-analyses, conference abstracts, editorials, commentaries and other non-original reports;
- Studies using non-plant-derived nanoparticles or non-green synthesized nanoparticles;
- Clinical or human studies;
- Studies not using an induced-PCOS rodent model;
- Non-therapeutic PCOS studies (e.g., etiology, diagnosis, or general background papers);
- Non-metallic/non-elemental nanoparticle platforms.

### Quality appraisals

To improve transparency in the interpretation of the included animal studies, a simplified manual appraisal was undertaken directly from the full texts of the five included studies. Reporting quality was appraised using selected ARRIVE-relevant items covering title and objectives, ethical approval, animal characteristics, experimental design, sample size, sample-size calculation, allocation procedures, housing and husbandry, outcome/statistical reporting, and safety or toxicity reporting. Risk of bias was appraised descriptively using selected SYRCLE-relevant domains, including sequence generation, baseline characteristics, allocation concealment, random housing, blinding, random outcome assessment, incomplete outcome data, selective outcome reporting, and other potential sources of bias. Consistent with the scoping nature of the review, these appraisals were used to improve transparency and contextualize the reliability of the evidence rather than to generate a full formal study-level quality score.

#### Quality appraisal of included studies

##### Reporting quality assessment

The outcome of the reporting quality assessment is displayed in Table 1. All five studies provided a clear title, stated the study objective, and reported the main experimental outcomes. Three studies (60%) reported ethical approval or a relevant approval number, whereas two studies (40%) did not. All five studies provided relevant information on the experimental animals, clearly described the experimental design and comparator structure, and reported the total number of animals used together with the sample size in each group. However, none of the studies explained how the sample size was determined. Three studies (60%) reported random grouping or random division of animals into the experimental groups, whereas two studies (40%) did not explicitly report allocation procedures. Housing and husbandry were clearly reported in three studies (60%), possibly sufficient in one study (20%), and clearly insufficient in one study (20%). All five studies clearly reported the principal procedures, outcomes, and statistical methods. No study clearly reported adverse events or a dedicated toxicity assessment within the main experimental design; instead, three studies (60%) provided limited or indirect safety information, while two studies (40%) did not report safety or toxicity. Overall, the included studies reported procedures and outcomes with reasonable clarity, but important methodological details relevant to internal validity and interpretability were incompletely reported.

**Table 1:** The reporting quality assessments of included articles based on selected ARRIVE-relevant items

Reference	1	2	3	4	5	6	7	8	9	10
(Alwan and Al-Saeed 2021)	2	2	2	2	2	0	1	2	2	1
(Alwan and Al-Saeed 2023)	2	2	2	2	2	0	1	2	2	1
(Akintola et al. 2024)	2	2	2	2	2	0	2	2	2	1
(Shaheen et al. 2024)	2	0	2	2	2	0	2	0	2	0
(Al-Bayati et al. 2024)	2	0	2	2	2	0	2	1	2	0
The percentage of “2” (%)	100	60	100	100	100	0	60	60	100	0
The percentage of “1” (%)	0	0	0	0	0	0	40	20	0	60
The percentage of “0” (%)	0	40	0	0	0	100	0	20	0	40

“2”: clearly sufficient; “1”: possibly sufficient; “0”: clearly insufficient, Items: 1, clear title, abstract, and research objective; 2, ethical approval or license number reported; 3, experimental animal characteristics reported; 4, experimental design and comparator groups clearly described; 5, total sample size and group sample size reported; 6, sample-size calculation explained; 7, randomization or allocation procedure reported; 8, housing and husbandry conditions reported; 9, experimental procedures, outcomes, and statistical methods clearly reported; 10, adverse events, safety, or toxicity reporting

##### Risk-of-bias assessment

**Fig. 3:**
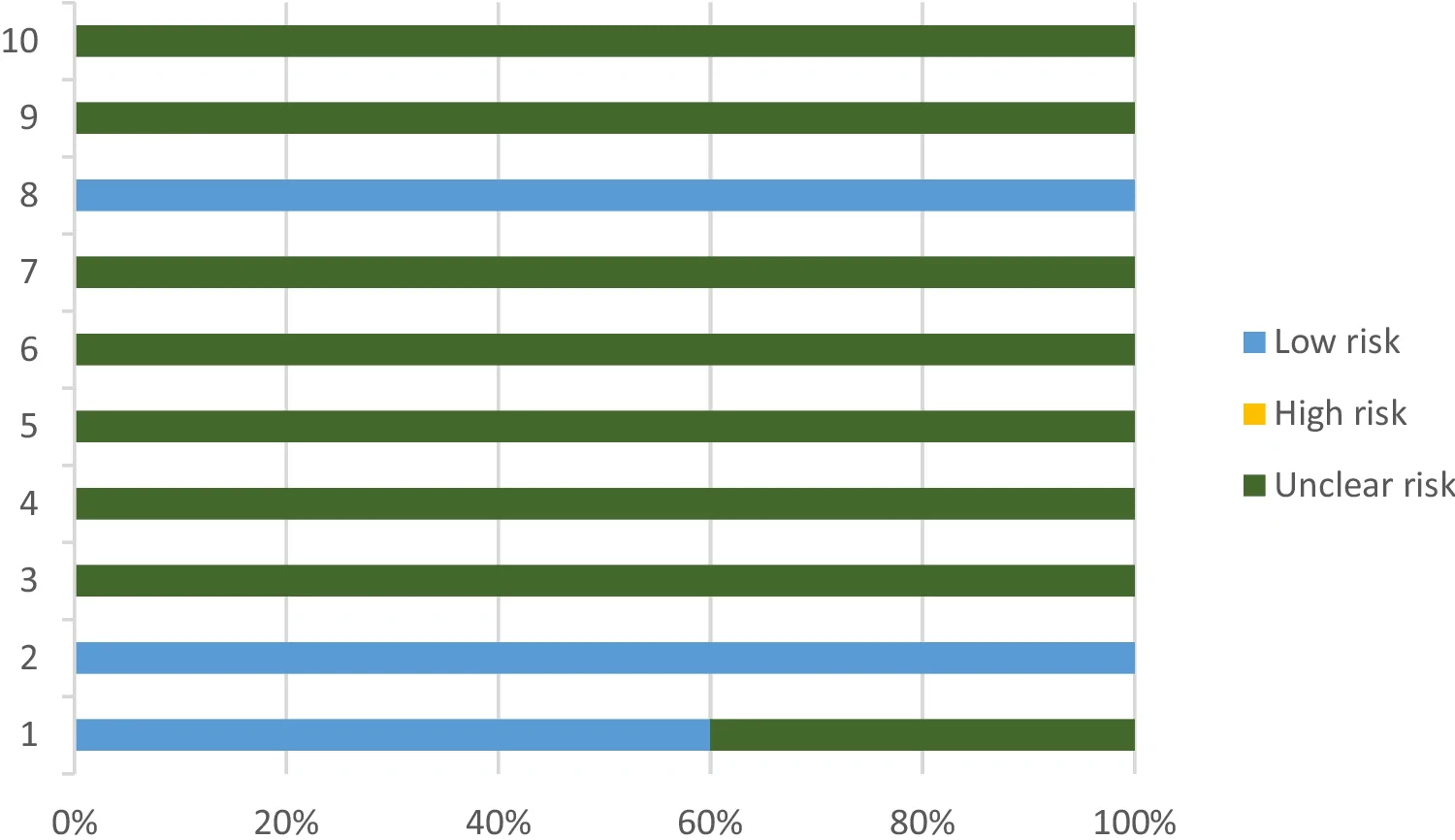
The risk-of-bias assessment of included studies based on selected SYRCLE-relevant domains. Note: 1, sequence generation (selection bias); 2, baseline characteristics (selection bias); 3, allocation concealment (selection bias); 4, random housing (performance bias); 5, blinding (performance bias); 6, random outcome assessment (detection bias); 7, blinding (detection bias); 8, incomplete outcome data (attrition bias); 9, selective outcome reporting (reporting bias); 10, other sources of bias (other)

The outcome of the risk-of-bias assessment is shown in Fig. 3. Sequence generation was judged as low risk in three studies (60%) and unclear in two studies (40%). Baseline characteristics were judged as low risk in all five studies (100%). Allocation concealment, random housing, performance blinding, random outcome assessment, detection blinding, selective outcome reporting, and other sources of bias were all judged as unclear in all or most studies because these methodological safeguards were insufficiently reported. Incomplete outcome data were judged as low risk in all five studies (100%). No domain was judged as high risk across the included studies. Overall, the pattern indicates that the main limitation was not necessarily demonstrated methodological weakness, but rather incomplete reporting of key study design safeguards.

### Data extraction and charting

A standardized charting form was used to record, for each included study:

- Bibliographic details: authors, year, and country.
- Nanoparticle attributes: plant source, synthesis method (plant extract/green method), metallic/elemental nanoparticle type, and reported characterization (size, morphology, UV–Vis peak, and additional analyses where reported).
- Experimental model: rodent species/strain, PCOS induction method, and intervention design.
- Dosing and administration: dose, route, and treatment duration.
- Comparators and controls: control groups and any standard-drug comparators where reported.
- Therapeutic outcomes: hormonal outcomes, ovarian histology/morphology, and metabolic/inflammatory/oxidative markers.
- Safety/toxicity: reported adverse effects or toxicity indicators where assessed.

### Data synthesis and reporting

Findings were synthesized narratively and summarized in tables and figures to map nanoparticle characteristics, therapeutic outcomes, and reported safety observations. Given the scoping nature of this review, a formal risk-of-bias assessment was performed, key methodological limitations and reporting gaps were described. The conduct and reporting were further guided by published methodological guidance for scoping reviews (Peters et al. 2015, 2020).

## Results

### Study selection

**Fig. 4:**
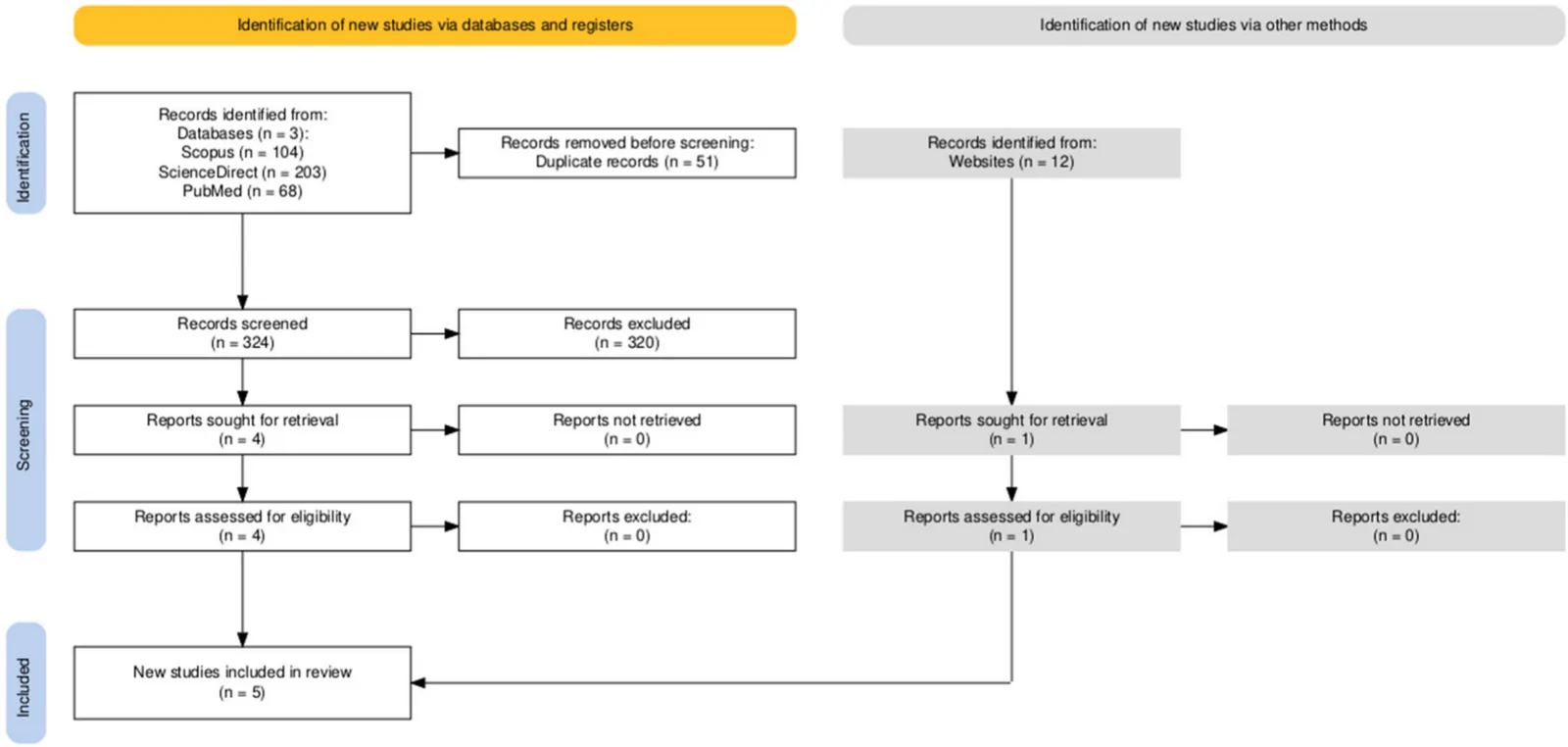
PRISMA-ScR flow diagram of study selection (Haddaway et al. 2022)

A PRISMA flow diagram summarizes the study selection process (Fig. 4). The database search yielded 375 records (Scopus = 104; ScienceDirect = 203; PubMed = 68). Following removal of 51 duplicates, 324 records underwent title and abstract screening, of which 320 were excluded. The remaining 4 reports were sought for retrieval, all of which were obtained (0 not retrieved) and assessed for full-text eligibility. No report from the database-search stream was excluded after full-text assessment, and all 4 studies were included. In the other methods stream, 12 records were identified, from which 1 report was sought for retrieval, successfully retrieved, assessed for eligibility, and included. Thus, a total of 5 studies were included in the review.

### Nanoparticle characterization

#### Morphology and particle-size characteristics

Nanoparticle performance is strongly influenced by physicochemical properties; therefore, the included preclinical studies reported morphology, particle-size metrics, and surface chemistry using complementary characterization techniques, including UV–visible spectroscopy, electron microscopy (SEM/FE-SEM/TEM), atomic force microscopy (AFM), Fourier-transform infrared (FTIR) spectroscopy, X-ray diffraction (XRD), and (where available) zeta potential analysis (Alwan and Al-Saeed 2021; Akintola et al. 2024; Al-Bayati et al. 2024).

Across studies, nanoparticles were predominantly described as spherical on electron microscopy. Cinnamon-mediated AgNPs were reported as smooth/spherical and below 100 nm, commonly ranging from 60 to 80 nm (Alwan and Al-Saeed 2021). *Fenugreek*-derived AgNPs were reported to range between 8.932 and 33.50 nm and were described as having an irregular spherical shape on FE-SEM (Al-Bayati et al. 2024). In the Corchorus olitorius SeNPs study, particle-size distribution was largely between 60 and 120 nm, while XRD reported a crystalline size of 14.81 nm, with microscopy indicating aggregated spherical particles (Akintola et al. 2024). In the Asparagus officinalis study, AFM analysis showed a mean particle diameter of 45.83 nm for Asp.NPs, while the average size increased to 278.4 nm after metformin loading; FTIR and AFM findings were consistent with successful metformin loading onto the nanocomposite (Shaheen et al. 2024).

Where both particle/aggregate size and XRD crystallite size were reported, crystallite size was smaller than the corresponding particle-size measure, consistent with primary crystallites existing within larger particles or aggregates; this was evident for SeNPs, with an XRD crystallite size of 14.81 nm versus a particle-size distribution of 60–120 nm, and for cinnamon AgNPs, with a Debye–Scherrer crystallite size of 36.4 nm versus an AFM size range of 60–80 nm (Alwan and Al-Saeed 2021; Akintola et al. 2024; Al-Bayati et al. 2024).

#### Zeta potential

Surface charge, expressed as zeta potential, is routinely reported as an indicator of colloidal stability, with potential implications for dispersion behavior and nanoparticle biological interactions in vivo. Where reported, cinnamon-derived AgNPs showed a zeta potential of approximately − 23 mV, consistent with moderate colloidal stability (Alwan and Al-Saeed 2023). In contrast, zeta potential was not explicitly reported for the *Corchorus olitorius* SeNP study, the *fenugreek*-derived AgNPs study, or the *Asparagus officinalis* nanocomposite study (Akintola et al. 2024; Al-Bayati et al. 2024; Shaheen et al. 2024). Comparative nanoparticle characterization parameters across the included preclinical studies are summarized in Table 2.

**Table 2:** Comparative summary of nanoparticle characterization parameters across included preclinical studies

Study	Plant source/nanoparticle	Methods reported	Size (reported)	Morphology	Zeta potential	Crystallinity/structural readout
(Alwan and Al-Saeed 2021)	*Cinnamomum zeylanicum* bark extract AgNPs	UV–Vis, SEM, AFM, FTIR, XRD, zeta potential	60–80 nm by AFM; below 100 nm by SEM	Smooth, spherical; minor aggregation noted	− 23.07 mV	XRD confirmed crystalline AgNPs; crystallite size 36.4 nm; UV–Vis peak 428 nm
(Alwan and Al-Saeed 2023)	*Cinnamomum zeylanicum* bark extract AgNPs	SEM, AFM	60–80 nm	Smooth, spherical	NR	No XRD or zeta potential reported
(Akintola et al. 2024)	*Corchorus olitorius* leaf extract SeNPs	EDX, FTIR, XRD, TEM, SEM, particle-size distribution	60–120 nm particle distribution	Aggregated, spherical particles	NR	XRD crystallite size 14.81 nm; FTIR showed phytochemical capping
(Shaheen et al. 2024)	*Fresh Asparagus officinalis* stem-derived AgNPs/asparagus nanocomposite	FTIR, AFM	Average diameter 45.83 nm for Asp.NPs; 278.4 nm after metformin loading	AFM nanoscale surface topography; shape not clearly stated in the text	NR	FTIR shifts supported AgNP formation and metformin loading; no XRD; AFM supported nanoscale particle measurements
(Al-Bayati et al. 2024)	*Trigonella foenum-graecum* aqueous extract AgNPs	UV–Vis, FE-SEM	8.932–33.50 nm	Irregular spherical	NR	UV–Vis peak 343 nm; no XRD reported

Abbreviations: *AgNPs*, silver nanoparticles; *SeNPs*, selenium nanoparticles; *AFM*, atomic force microscopy; *EDX*, energy-dispersive X-ray analysis; *FE-SEM*, field-emission scanning electron microscopy; *FTIR*, Fourier-transform infrared spectroscopy; *NR*, not reported; *SEM*, scanning electron microscopy; *TEM*, transmission electron microscopy; *UV–Vis*, ultraviolet–visible spectroscopy; *XRD*, X-ray diffraction

### Comparative therapeutic efficacy of plant-derived nanoparticles in PCOS management

#### Therapeutic efficacy outcomes

Despite differences in materials, the included studies generally reported beneficial effects on PCOS-related outcomes (Shi et al. 2024). These therapeutic effects are summarized below according to their primary physiological targets.

#### Hormonal regulation and ovarian function

Across included studies, plant-derived metallic nanoparticles improved PCOS-relevant outcomes. Cinnamon-AgNPs significantly decreased testosterone and increased serum FSH and progesterone, while LH showed an apparent decline versus PCOS controls without statistically significant differences, alongside improved estrous cyclicity and fertility outcomes (Alwan and Al-Saeed 2021). The Corchorus olitorius SeNPs study reported higher estrogen and progesterone with depleted LH and FSH, together with enzymatic modulation enhanced ovarian aromatase activity and reduced 17β-HSD activity (Akintola et al. 2024). The *fenugreek*-AgNPs study reported endocrine improvements, including increased FSH, progesterone, and estrogen, with decreases in LH and prolactin versus PCOS controls (Al-Bayati et al. 2024). By contrast, the Asparagus officinalis study focused primarily on immunological endpoints rather than endocrine markers, and is therefore considered mainly within the inflammatory-response domain (Shaheen et al. 2024).

#### Anti-inflammatory and antioxidant effects

Inflammation is increasingly described as a central feature of PCOS, with elevated pro-inflammatory cytokines reported in both clinical and experimental contexts; when mode-of-action evidence endpoints are included, rodent studies commonly quantify cytokines and selected oxidative-stress response markers such as Nrf2 (Alwan and Al-Saeed 2023; Panchpuri et al. 2026). Inflammatory and antioxidant outcomes were reported in three included studies. In an estradiol-valerate PCOS rat model, Cinnamomum zeylanicum–derived AgNPs significantly reduced serum TNF-α, IL-6, and IL-18 compared with untreated PCOS animals (Alwan and Al-Saeed 2023).

In a letrozole-induced PCOS model, *Corchorus olitorius*–mediated SeNPs lowered TNF-α expression and restored MCP-1 toward control levels in neuroendocrine and reproductive tissues, alongside increased Nrf2 expression in hypothalamic and ovarian tissues after treatment (Akintola et al. 2024). In another letrozole-induced PCOS rat study, both fresh Asparagus officinalis stem-derived nanocomposite (Asp.NPs) and metformin-loaded Asp.NPs significantly decreased serum IL-10 and IL-17 relative to untreated PCOS rats after 28 days of oral treatment (Shaheen et al. 2024). By contrast, the *fenugreek*-AgNPs study focused on endocrine outcomes (LH, FSH, prolactin, progesterone, estrogen) and did not report inflammatory cytokines or oxidative-stress markers (Al-Bayati et al. 2024).

Overall, the biomarker-based evidence is supportive but incomplete, as broader cytokine panels and confirmatory molecular analyses were not consistently undertaken across studies (Alwan and Al-Saeed 2023).

### Toxicity and safety of plant-derived nanoparticles in PCOS models

Safety evidence in preclinical nanomedicine often remains uneven and short-term, and translation is constrained by the need for clearer biocompatibility, toxicity, and long-term safety evaluation, alongside more robust in vivo and clinical evidence (Muthukumaran and Shanmugam 2024; Sharma et al. 2025).

Across the included PCOS rodent studies, dose selection and short-term observations suggested no observable toxicity at the administered doses and treatment durations: the Corchorus SeNPs study reported that 1 mg/kg was an effective and non-toxic dose (Akintola et al. 2024); the EV-induced cinnamon AgNPs study noted that AgNP safety had been evidenced in prior work (Alwan and Al-Saeed 2023); the cinnamon AgNP fertility study recorded 0 deaths in the AgNP-treated group (Alwan and Al-Saeed 2021); and the *fenugreek* AgNP study primarily reported endocrine endpoints after 30-day oral dosing and serum hormone assays (Al-Bayati et al. 2024). The *Asparagus officinalis* nanocomposite study primarily reported immune biomarkers after 28 days of oral dosing and did not report a dedicated toxicity evaluation, limiting conclusions on safety beyond the measured cytokine outcomes (Shaheen et al. 2024).

Table 3 provides a consolidated summary of the study models, the dose and route of administration, and the key therapeutic outcomes.

**Table 3:** Summary of study models, dose and route, and key therapeutic outcomes across included preclinical studies on plant-derived nanoparticles for PCOS management

Study (year)	Plant source and nanoparticle	PCOS model (animal, induction)	Dose and route	Key therapeutic outcomes
(Alwan and Al-Saeed 2021)	Cinnamomum zeylanicum (cinnamon) bark extract-metallic nanoparticles (silver) (biosynthesized/green synthesis)	Rat (estradiol valerate (EV)-induced PCOS)	3.53 mg/kg body weight, oral; 30 days	AgNP treatment increases serum FSH (follicle stimulating hormone) and progesterone levels and significantly decreases E2 and testosterone levels. Improve the estrus cycle and fertility rate compared with the PCOS group. Fertility rate reported as 100% in the treated group when compared to the PCOS group
(Alwan and Al-Saeed 2023)	Cinnamomum zeylanicum (cinnamon) bark extract-metallic nanoparticles (silver) (biofabricated/green synthesis)	Rat (EV-induced PCOS)	3.53 mg/kg, oral; 30 days	Significant reductions in TNF-α, IL-6, IL-18 in serum (toward control levels) alongside improvement in endocrine markers consistent with the study
(Akintola et al. 2024)	Corchorus olitorius leaf extract-selenium nanoparticles (green synthesis)	Rat (letrozole-induced PCOS + L-NAME-induced hypertension)	1 mg/kg, oral; 35 days	Increased estrogen and progesterone with decreases in LH, FSH, and Testosterone. Reduction in ovarian steroidogenic enzymes (aromatase, 17β-HSD); inflammatory markers decreased (TNF-α and MCP-1) with increased Nrf2
(Shaheen et al. 2024)	*Asparagus officinalis* stem extract-silver nanoparticles/green asparagus nanocomposite; also evaluated as metformin-loaded nano-asparagine complex	Rat (letrozole-induced PCOS female white rats)	Oral, 28 days; Asp.NPs 300 mg/kg; metformin-loaded Asp.NPs 300 mg/kg	Asp.NPs and metformin-loaded Asp.NPs lowered IL-10 and IL-17 relative to the untreated PCOS group
(Al-Bayati et al. 2024)	Trigonella foenum-graecum aqueous extract-silver nanoparticles (green synthesis)	Rat (letrozole-induced PCOS; 1 mg/kg induction)	Nano-*fenugreek* extract 2 mg/kg, oral; 30 days	Increased FSH, progesterone and estrogen; decreased LH and prolactin relative to the untreated PCOS group

## Discussion

This scoping review synthesized recent preclinical evidence on plant-mediated metallic/elemental nanoparticles for the management of polycystic ovary syndrome (PCOS) in rodent models, summarizing the key outcomes commonly evaluated across studies. This review was conducted using established scoping review methodology and reporting guidance (Arksey and O'Malley 2005; Levac et al. 2010; Tricco et al. 2018; Peters et al. 2020). The available evidence was limited to five experimental rat studies. Most of these studies prioritized endocrine outcomes and ovarian function indices, as well as selected pathway-related markers where applicable (Alwan and Al-Saeed 2021, 2023; Akintola et al. 2024; Al-Bayati et al. 2024; Shaheen et al. 2024). In these studies, endocrine and ovarian outcomes were commonly reported, whereas inflammatory and antioxidant signaling markers were assessed in only a subset of the included studies.

Across the included experiments, PCOS was induced using either estradiol valerate (EV) or letrozole protocols, and treatments were administered orally for approximately 28—35 days, depending on the study design (Alwan and Al-Saeed 2021, 2023; Akintola et al. 2024; Al-Bayati et al. 2024; Shaheen et al. 2024). Two studies used an EV-induced PCOS model and evaluated cinnamon-derived silver nanoparticles (3.53 mg/kg) for 30 days (Alwan and Al-Saeed 2021, 2023). Letrozole-induced PCOS was used in the Corchorus-derived selenium nanoparticle study, the *fenugreek*-derived silver nanoparticle study, and the *Asparagus officinalis* nanocomposite study (Akintola et al. 2024; Al-Bayati et al. 2024; Shaheen et al. 2024). The use of letrozole in rodent PCOS induction is well established in the broader animal-model literature, where aromatase inhibition is used to generate a PCOS-like endocrine and ovarian phenotype (Kafali et al. 2004; Xu et al. 2020).

Nanoparticle characterization was reported because physicochemical properties (including size, morphology, crystallinity, and surface chemistry) can influence dispersion, tissue interaction, and biological effects in vivo, and such reporting is a core expectation in nanomedicine studies (Blanco et al. 2015; Mitchell et al. 2021). In addition, green synthesis using plant extracts is widely described as providing reducing and capping agents that influence particle formation and stability, making synthesis conditions and characterization particularly important for reproducibility (Iravani 2011; Kirubakaran et al. 2025). In this evidence set, morphology was predominantly described as spherical or near-spherical on microscopy, although aggregation or irregularity was noted in some formulations depending on synthesis conditions (Alwan and Al-Saeed 2021; Akintola et al. 2024; Al-Bayati et al. 2024). Cinnamon-mediated AgNPs were reported as < 100 nm, commonly 60–80 nm, while *fenugreek*-derived AgNPs were reported as 8.932–33.50 nm with an irregular spherical shape (Alwan and Al-Saeed 2021; Al-Bayati et al. 2024). In the Corchorus SeNP study, particle-size distribution was largely 60–120 nm, while XRD reported a smaller crystallite size of 14.81 nm, consistent with primary crystallites occurring within larger particles or aggregates (Akintola et al. 2024). In the asparagus-derived nanocomposite study, AFM showed an average particle diameter of 45.83 nm for Asp.NPs, which increased to 278.4 nm after metformin loading, while FTIR shifts were interpreted as evidence of successful loading of metformin onto the nanocomposite (Shaheen et al. 2024).

With respect to endocrine outcomes, most included studies reported improvements in PCOS-relevant hormones following nanoparticle treatment compared with untreated PCOS controls (Alwan and Al-Saeed 2021; Akintola et al. 2024; Al-Bayati et al. 2024). In the EV-induced PCOS model, cinnamon-derived AgNPs increased serum FSH and progesterone and decreased estradiol (E2) and testosterone, with reported improvement in the estrous cycle and fertility rate relative to the PCOS group (Alwan and Al-Saeed 2021). In the letrozole-induced model, Corchorus-derived SeNPs were reported to increase estrogen and progesterone while decreasing LH, FSH, and testosterone, alongside modulation of ovarian steroidogenic enzymes, including aromatase and 17β-HSD (Akintola et al. 2024). Similarly, *fenugreek*-derived AgNPs were associated with increased FSH, progesterone, and estrogen and decreased LH and prolactin relative to PCOS controls (Al-Bayati et al. 2024). By contrast, the asparagus-derived nanocomposite study focused primarily on immune endpoints and formulation-related findings rather than direct endocrine measurements, and is therefore more appropriately interpreted within the inflammatory-response domain (Shaheen et al. 2024).

Inflammation and oxidative-stress signaling are increasingly recognized as contributors to PCOS features, and animal studies may include cytokines and antioxidant-response markers where these outcomes are assessed (Gonzalez 2012; Murri et al. 2013). In this review, inflammatory or immune-related outcomes were reported in three studies, indicating that pathway-related reporting was present but not uniform across the included evidence (Alwan and Al-Saeed 2023; Akintola et al. 2024; Shaheen et al. 2024). Cinnamon-mediated AgNPs were reported to reduce pro-inflammatory cytokines, with decreases in TNF-α, IL-6, and IL-18 compared with PCOS controls (Alwan and Al-Saeed 2023). In the Corchorus SeNP study, reductions in TNF-α and MCP-1 were reported alongside increased Nrf2 expression in hypothalamic and ovarian tissues, consistent with activation of antioxidant-response signaling within the measured endpoints (Akintola et al. 2024). In the asparagus-derived nanocomposite study, Asp.NPs and metformin-loaded Asp.NPs decreased IL-10 and IL-17 relative to untreated PCOS animals (Shaheen et al. 2024).

Taken together, the mechanistic signals reported across the included studies suggest that plant-mediated nanoparticles may act through three related routes in experimental PCOS. First, some formulations appeared to influence steroidogenic balance, as shown by restoration of aromatase activity, reduction of 17β-HSD activity, and improvement in circulating reproductive hormones in the Corchorus SeNP study, together with normalization of FSH, progesterone, estrogen, testosterone, LH, or prolactin in the cinnamon and *fenugreek*-AgNPs studies (Alwan and Al-Saeed 2021; Akintola et al. 2024; Al-Bayati et al. 2024). Second, several studies support an anti-inflammatory mechanism, with reductions in TNF-α, IL-6, IL-18, MCP-1, IL-17, and IL-10 after treatment, depending on the biomarker panel used (Alwan and Al-Saeed 2023; Akintola et al. 2024; Shaheen et al. 2024). Third, antioxidant-response modulation was directly suggested only in the Corchorus SeNP study, in which Nrf2 expression increased after treatment (Akintola et al. 2024). In addition, the asparagus study supports a formulation-related contribution rather than a fully resolved mechanism, because FTIR and AFM findings supported successful metformin loading, and both Asp.NPs and metformin-loaded Asp.NPs reduced IL-10 and IL-17 relative to untreated PCOS rats. These findings suggest that the nanocomposite formulation may have contributed to the observed immune-marker effects of the loaded treatment (Shaheen et al. 2024). However, none of the included studies directly quantified insulin-signaling endpoints such as fasting insulin, glucose homeostasis, HOMA-IR or downstream molecular targets. Consequently, the current evidence more directly supports the effects of nanoparticles on steroidogenesis, inflammatory cytokines and antioxidant-response pathways than on insulin signaling itself (Alwan and Al-Saeed 2021, 2023; Akintola et al. 2024; Al-Bayati et al. 2024; Shaheen et al. 2024). Therefore, any link to improved insulin resistance should be interpreted as a broader pathophysiological inference in PCOS, rather than as a mechanism that has been demonstrated directly in the nanoparticle studies included in this review (Gonzalez 2012; Murri et al. 2013).

Finally, although the reported endocrine and inflammatory improvements are promising, the evidence remains preclinical and short-term, and safety reporting was not standardized across studies. These findings should also be interpreted in light of the simplified quality appraisal, which showed incomplete reporting of randomization, blinding, sample-size justification, and safety assessment across the included studies (Alwan and Al-Saeed 2021, 2023; Akintola et al. 2024; Al-Bayati et al. 2024; Shaheen et al. 2024). No study provided a comprehensive long-term toxicology profile or bioaccumulation assessment sufficient for firm safety conclusions beyond the reported durations and endpoints, and this limitation is consistent with broader nanomedicine guidance emphasizing the need for rigorous characterization, safety assessment, and reproducible design principles prior to clinical translation (Blanco et al. 2015; Mitchell et al. 2021). In parallel, while plant extracts alone are widely used and discussed for PCOS, limitations such as poor solubility, instability, and variable bioavailability are frequently noted, providing a rationale for exploring nanoparticle-enabled formulations (Lopresti 2018; Kwon et al. 2020). The absence of a dedicated toxicity evaluation in the asparagus-derived nanocomposite study further illustrates how uneven safety reporting remains across this evidence base (Shaheen et al. 2024). Future work should therefore prioritize formulation standardization, including reporting of synthesis conditions and stability, confirmation of pathway-related effects using harmonized outcome sets, and longer-duration safety assessment, followed by rigorously designed clinical trials before clinical application can be recommended (Mitchell et al. 2021; Muthukumaran and Shanmugam 2024; Butt et al. 2025; Kirubakaran et al. 2025).

### Comparison with existing nanomedicine reviews

In comparison to broader nanomedicine reviews on PCOS, this scoping review takes a more specific approach by limiting the scope of the evidence map to plant-mediated metallic/elemental nanoparticles synthesized using green methods and evaluated in induced PCOS rodent models (Muthukumaran and Shanmugam 2024; Shi et al. 2024; Sharma et al. 2025).

By contrast, more recent, broader reviews have discussed a much wider range of nanotechnology-based approaches. These include polymeric systems, liposomes, micelles, metal and metal oxide nanoparticles, targeted ligands, extracellular vesicles and diagnostic nanoplatforms. Often, the evidence base is not restricted to plant-mediated systems or preclinical therapeutic rodent studies (Muthukumaran and Shanmugam 2024; Shi et al. 2024; Yang et al. 2024; Vijayakumar et al. 2025).

This narrower scope enabled a more direct comparison of the plant source, nanoparticle type, endocrine outcomes, inflammatory markers, and reported safety observations across the included studies (Alwan and Al-Saeed 2021, 2023; Akintola et al. 2024; Al-Bayati et al. 2024; Shaheen et al. 2024). However, it also revealed that the current evidence base for this specific subfield remains limited, with only a small number of eligible animal studies meeting these stricter criteria (Muthukumaran and Shanmugam 2024; Shi et al. 2024; Sharma et al. 2025).

### Clinical implications

The findings of this review suggest that plant-mediated metallic/elemental nanoparticles may be useful in the treatment of PCOS, as the included rodent studies reported improvements in reproductive hormone profiles, ovarian morphology, estrous cyclicity, fertility-related outcomes, and selected inflammatory or immune-related markers (Alwan and Al-Saeed 2021, 2023; Akintola et al. 2024; Al-Bayati et al. 2024; Shaheen et al. 2024). These findings are clinically relevant, as current PCOS management relies mainly on lifestyle intervention and pharmacological therapies targeting symptoms, such as metformin, combined oral contraceptives, and ovulation-inducing agents, rather than nanoparticle-based therapeutic platforms (Teede et al. 2023; Shi et al. 2024). However, the current evidence should be interpreted with caution, as it is entirely preclinical and based on a small number of heterogeneous rodent studies. The evidence is also limited by the short duration of treatment and the incomplete reporting of long-term safety data (Muthukumaran and Shanmugam 2024; Shi et al. 2024; Sharma et al. 2025). Clinical translation is further limited by challenges in scaling green-synthesis approaches while preserving reproducibility, batch-to-batch consistency, and standardized nanoparticle characteristics, as well as regulatory requirements for clinical-grade manufacturing and safety evaluation (Herdiana et al. 2022; Ayub et al. 2025; Lakhani et al. 2025). Accordingly, these findings should be viewed as promising preclinical signals rather than as evidence ready for clinical implementation (Teede et al. 2023; Muthukumaran and Shanmugam 2024; Shi et al. 2024).

### Future research directions

Future studies should prioritize standardization of green synthesis and nanoparticle characterization, particularly particle size distribution, morphology, crystallinity, surface chemistry, zeta potential, and colloidal stability, because these properties influence reproducibility and biological behavior of the formulations (Blanco et al. 2015; Mitchell et al. 2021; Shi et al. 2024). Preclinical designs should also use more harmonized PCOS outcome sets and appropriate comparators, including untreated PCOS controls, plant extract alone and standard therapies, to clarify whether observed effects arise from the nanoparticle system, the phytochemical source, or both (Paixão et al. 2017; Shi et al. 2024).

Longer-duration safety assessment, together with dose–response, pharmacokinetic, and biodistribution studies, will be needed before translation can be justified (Blanco et al. 2015; Mitchell et al. 2021; Muthukumaran and Shanmugam 2024). Once these preclinical gaps are addressed, well-designed clinical trials will be required to confirm safety and therapeutic efficacy in humans (Teede et al. 2023; Shi et al. 2024).

## Conclusion

This scoping review identified limited but promising preclinical evidence on plant-mediated metallic/elemental nanoparticles for PCOS in rodent models. Across the five included studies, these interventions were associated with improvements in key PCOS-related outcomes, including normalization of reproductive hormone profiles, restoration of estrous cyclicity and/or fertility-related measures, improvement in ovarian histomorphology, and reductions in selected inflammatory or immune-related markers where assessed. However, the evidence base remains small and heterogeneous, and clinical translation is constrained by limited standardization of nanoparticle characterization and incomplete long-term safety reporting.

## Supplementary Information

Below is the link to the electronic supplementary material.

ESM 1 (DOCX 27.2 KB)

## Data Availability

All source data for this work (or generated in this study) are available upon reasonable request.
