# Supplementary material for: Exploring the role of medicinal plants and nanotechnology in the management of polycystic ovarian syndrome (PCOS): a scoping review
Source: Naunyn Schmiedebergs Arch Pharmacol. 2026 May 14;399(11):16619–33. doi: 10.1007/s00210-026-05425-3 (PMC13503477; doi:10.1007/s00210-026-05425-3)
Supplement: Supplementary file 1 [file 210_2026_5425_MOESM1_ESM.docx]

**Supplementary Table S1. Full database-specific search strategies**

Search terms were entered manually in the advanced search interfaces using Boolean operators (AND, OR), parentheses for term grouping, quotation marks for phrase searching, and the wildcard symbol (*) where applicable. Searches were restricted to English-language publications and a year range of 2015–2025.

| **Database** | **Search string** | **Applied limits/ filters** | **Notes** |
| --- | --- | --- | --- |
| ScienceDirect | (PCOS OR "polycystic ovary syndrome") AND (nanoparticle OR nanoparticles) AND ("green synthesis" OR biogenic OR phytosynthesis OR "plant-mediated synthesis") | Year range: 2015–2025 Language: English | Advanced search |
| Scopus | ("polycystic ovary syndrome" OR "polycystic ovarian syndrome" OR PCOS) AND (nanoparticle* OR nanotechnology OR nanomedicine) AND (human* OR women OR patient* OR animal* OR rat* OR mouse* OR rodent*) | Year range: 2015–2025 Language: English | Advanced search |
| PubMed | ((polycystic ovary syndrome OR polycystic ovarian syndrome OR PCOS) AND (nanoparticles* OR nanotechnology OR nanomedicine) AND (human* OR women OR animal* OR rat* OR mouse* OR rodent*)) | Filters: 2015–2025 Language: English | Advanced search |
| Google Scholar/Publish or Perish | "Polycystic ovary syndrome" rat nanoparticles "plant extract" "green method" | Year range: 2015–2025 Language: English | Search conducted via Harzing’s Publish or Perish software |
